# Supplementary material for: Dishevelled has a YAP nuclear export function in a tumor suppressor context-dependent manner
Source: Nat Commun. 2018 Jun 12;9:2301. doi: 10.1038/s41467-018-04757-w (PMC5997650; doi:10.1038/s41467-018-04757-w)

SUPPLEMENTARY INFORMATION

Dishevelled has a YAP nuclear export function in a tumour suppressor context-dependent manner

Yoonmi Lee et al.

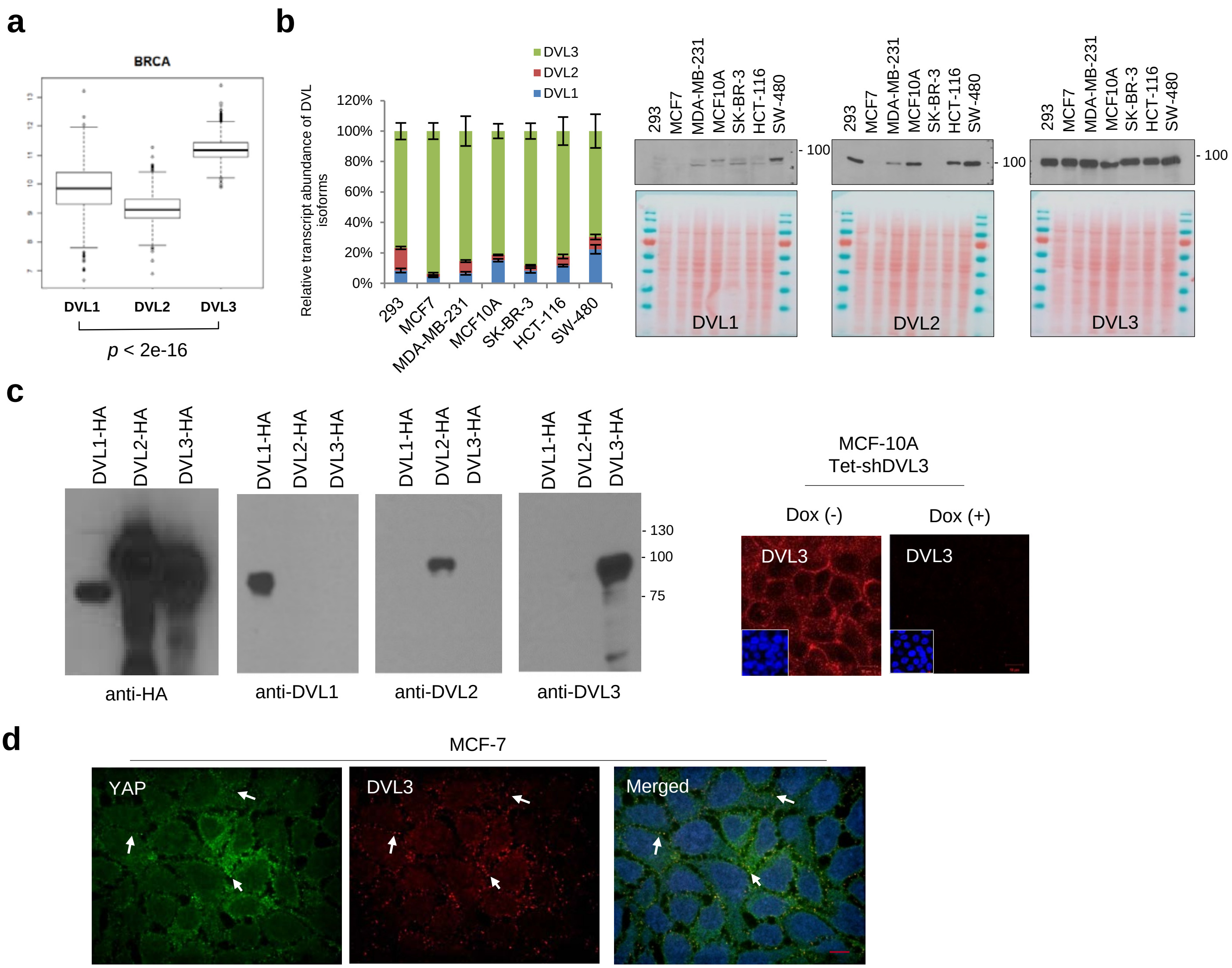

Supplementary Figure 1. Human cancer cells mainly express the DVL3 isoform**.** (**a**) Transcript abundance of DVL isoforms in breast cancer samples. The transcriptome data of breast cancer (BRCA) samples was downloaded from TCGA, and the transcript abundance of DVL isoforms was analyzed. Statistical significance was determined by One-way ANOVA. (**b**) Transcripts and protein abundance of DVL isoforms from various human cancer cells. Relative transcripts abundance from various cell lines was detected by quantitative RT-PCR using isoform specific primers having about 80 bp amplification target sequences (left panel). Ten µg of each cell lysate was subjected to immunoblot analysis for DVL isoforms. The films were developed at the same exposures to compare relative protein abundance of DVL isoforms (upper panels), and protein loading of each blot was validated with Ponceau stain (lower panels). (**c**) The specificity of antibody against DVL isoforms was determined with tagged expression vectors. HA-tagged DVL1, DVL2, and DVL3 were transfected into HEK 293 cells and the cell lysates were subjected to immunoblot analysis with anti-DVL1, anti-DVL2, anti-DVL3, and anti-HA antibodies. Specificity of DVL3 antibody for immunofluorescence was validated using inducible knockdown of DVL3 in MCF-10A cells (right panels). Inset; DAPI. (**d**) Confocal images of endogenous YAP (green) and DVL3 (red) in serum starved MCF-7 cells. Nuclear staining with TOPRO3 (blue) is shown in merged image. Arrows indicate co-localized foci. Scale bar, 10 µm.

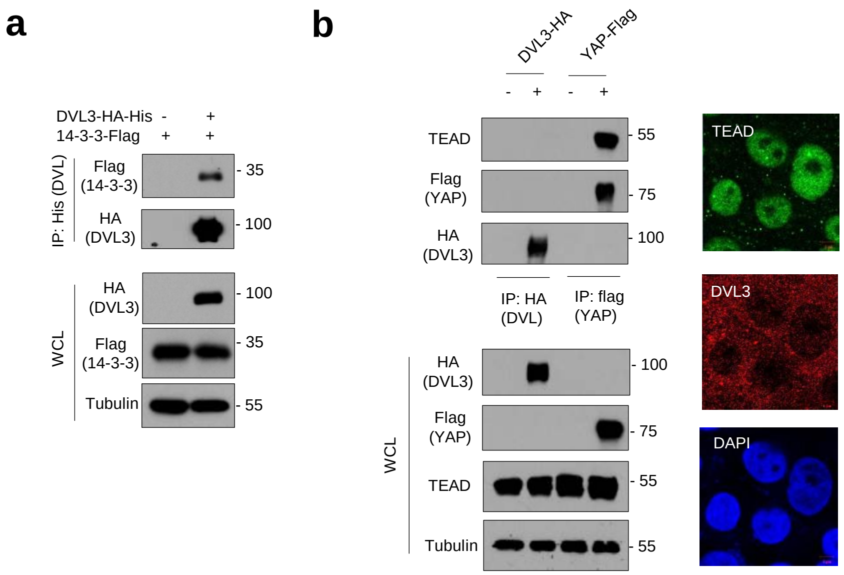

Supplementary Figure 2. DVL3 interacts with 14-3-3, but not with TEAD transcription factor**.** (**a**) 293A cells were transfected with His-HA-tagged DVL3 and vector control (-) or flag-tagged 14-3-3. Interaction between DVL and 14-3-3 was determined following immunoprecipitation (IP) with Ni-NTA beads and immunoblotting with anti-flag. Whole cell lysate (WCL) serves as input abundance for IP. (**b**) DVL3 does not interact with TEAD transcription factor, while it binds to YAP which served as positive control. 293A cells were transfected with HA-tagged DVL3 and vector control (-) or flag-tagged YAP. Interaction between DVL and endogenous TEAD or overexpressed YAP were determined following immunoprecipitation (IP) with anti-HA antibody for DVL3 or anti-Flag for YAP and immunoblotting with anti-flag and anti-pan-TEAD. Whole cell lysate (WCL) serves as input abundance for IP (left panels). Confocal images of endogenous pan-TEAD (green) and DVL3 (red) in serum-starved 293A (right panels). Nuclear staining with DAPI (blue); Scale bar, 5 µm.

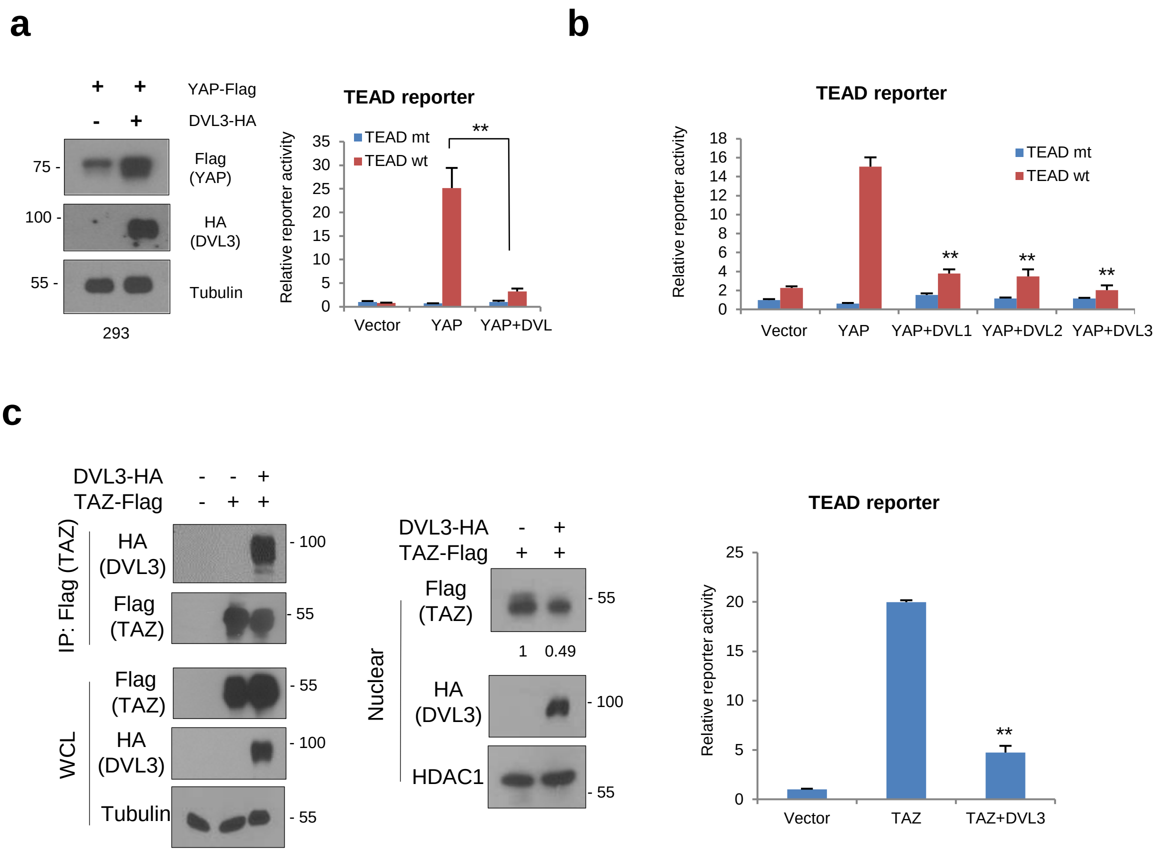

Supplementary Figure 3. DVL suppresses nuclear YAP. (**a**) YAP was co-transfected with DVL3 into HEK 293 cells, and protein abundance (left panels) and TEAD reporter activity were determined. Data of reporter assay was normalized to control empty vector (-) and presented as mean ± SD from triplicate experiments (right panel). (**b**) TEAD reporter construct was transfected with YAP in combination with DVL isoforms, and the relative reporter activity was determined. (**c**) DVL3 interacts with TAZ and suppresses nuclear TAZ activity. The 293 cells were transfected with HA-tagged DVL3 and vector control (-) or flag-tagged TAZ. Interaction between DVL3 and TAZ were determined with immunoprecipitation analysis (left panels). The TAZ was transfected with vector control (-) or DVL3, and nuclear TAZ abundance was measured by immunoblot analysis (middle panels). HDAC1 served as loading control of nuclear lysates. The TEAD reporter was co-transfected with vector or TAZ or TAZ in combination of DVL3, and the relative reporter activities were measured (right panel).

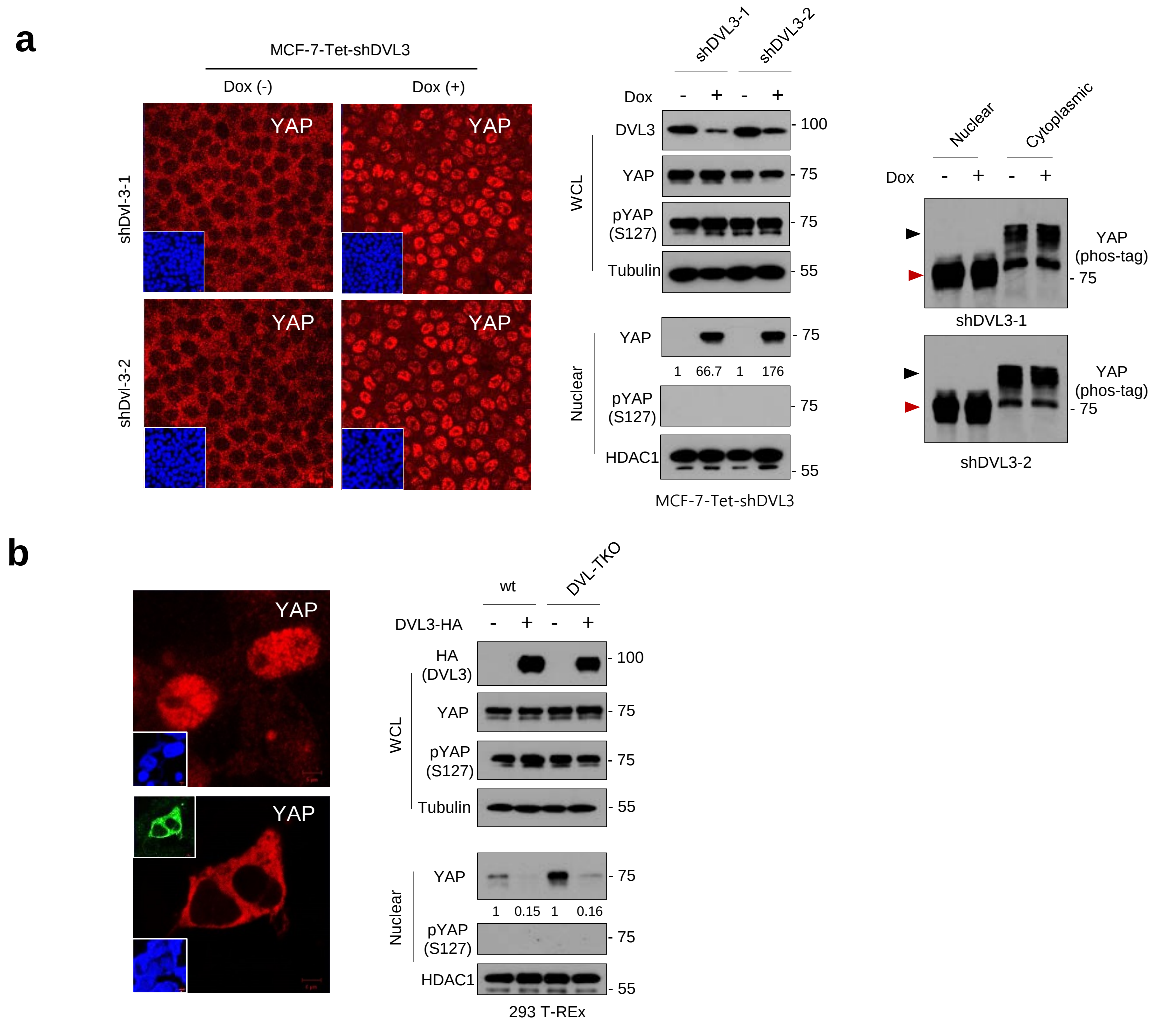

Supplementary Figure 4. DVL regulates nuclear YAP abundance. (**a**) Knockdown of DVL3 increased nuclear YAP abundance in MCF-7 cells. Inducible knockdown of DVL3 induced YAP nuclear accumulation in serum-starved confluent MCF-7 cells, observed by confocal immunofluorescence microscopy. Nuclear staining with DAPI is shown below each panel (left panels). Scale bar, 10 μm. The MCF-7 cells expressing inducible shRNA against DVL3 were cultured under confluent condition and then serum starved for 16 hr before harvest in the absence or presence of doxycycline (Dox) for 48 hr, and the whole cell lysaste (WCL) and nuclear protein were analyzed with immunoblot analysis to determine endogenous DVL3 and YAP abundance (middle panels). The relative YAP abundance of each shRNA was measured with ImageJ. Phosphorylation status of endogenous nuclear and cytoplasmic YAP was determined by mobility shift on a phos-tag gel (right panels). Red arrows indicate active YAP. (**b**) Re-introduction of wt DVL3 into DVL-TKO 293 T-REx cells rescues YAP subcellular localization to cytoplasmic space. The DVL-TKO cells were transfected with HA-tagged DVL3 and YAP localization was determined by confocal microscopy (left panels). Upper inset, HA (DVL3); lower inset, DAPI, Scale bar, 5 μm. The wt and DVL-TKO cells were transfected with HA-tagged DVL and YAP abundance from whole cell lysate (WCL) and nuclear fraction was determined by immunoblot analysis (right panels).

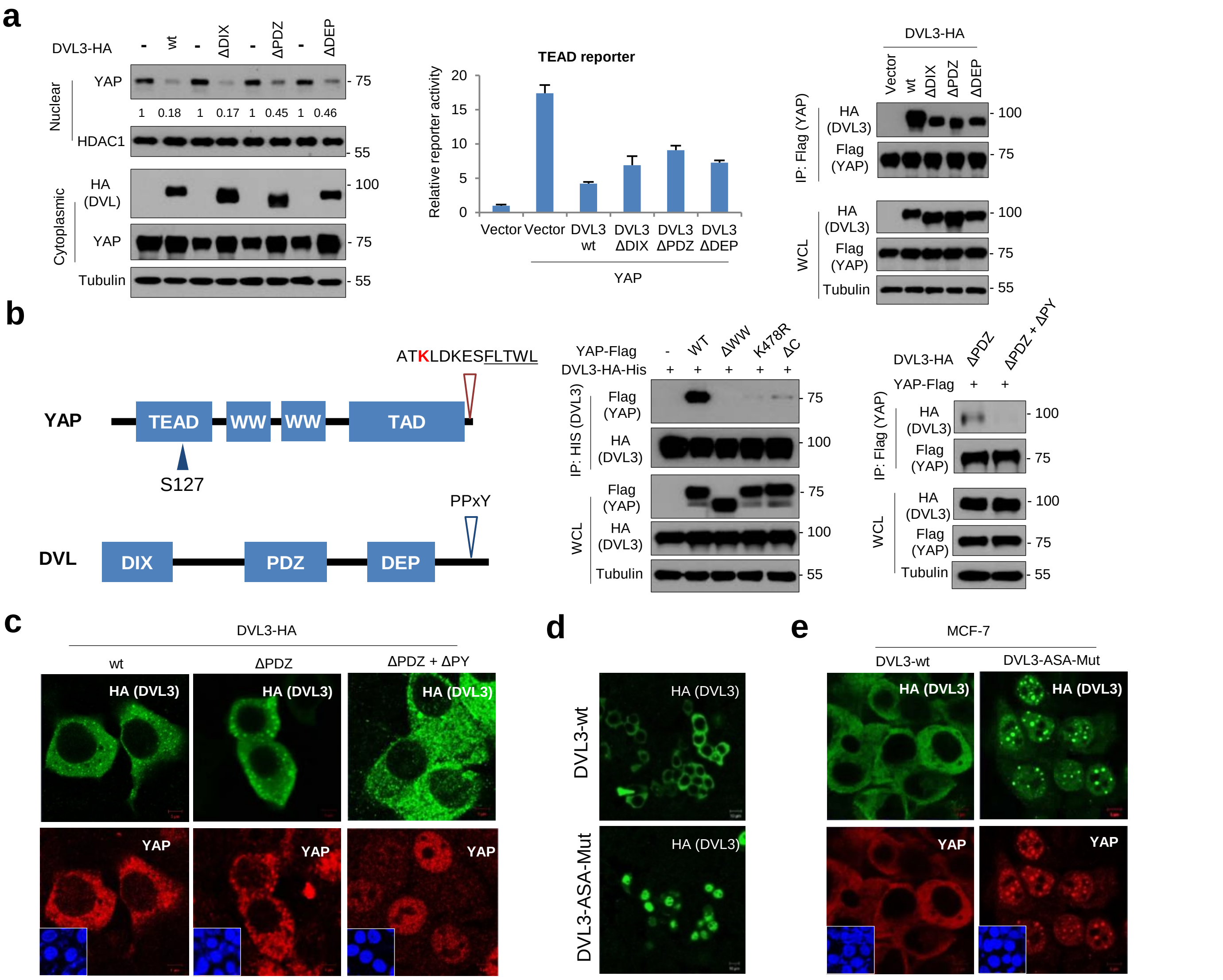

Supplementary Figure 5. Conserved domains of DVL are not responsible for nuclear YAP activity. (**a**) YAP interacts with deletion mutants of DVL. The 293 cells were transfected with flag-tagged YAP with control (-) or various deletion mutants of conserved domain of DVL, and nuclear YAP abundance (left panels) and TEAD transcriptional activities (middle panel) were determined by immunoblot analysis and reporter assay. The 293 cells were co-transfected with the indicated HA-tagged DVL3 or mutant constructs and flag-tagged YAP. Cell lysates were subjected to anti-flag immunoprecipitation, and the presence of DVL was assessed by anti-HA immunoblotting (right panels). (**b**) C-terminus/WW domain of YAP and PY motif/PDZ domain of DVL are required for YAP-DVL interaction. Schematic representation of the conserved domains of YAP and DVL (left panels). The PDZ-binding motif and monomethylation site of lysine 494 were indicated as underlined and red, respectively. The 293 cells were co-transfected with the indicated mutant constructs and flag-tagged YAP. Cell lysates were subjected to anti-His (middle panels) or anti-flag (right panels) immunoprecipitation and subsequent immunoblot analysis. (**c**) Deletion of PY motif and PDZ domain lacks YAP nuclear export. The 293 cells were transfected with wt or deletion mutants of DVL3, then DVL and YAP localization were determined by confocal microscopy. Inset, DAPI; Scale bar, 5 μm. (**d**) Mutational inactivation of NES in DVL3 increased nuclear retention of DVL. The 293 cells were transfected with wt or ASA-mut of DVL3 and observed by confocal immunofluorescence microscopy. Scale bar, 10 μm. (**e**) The MCF-7 cells were stably transfected with wt or ASA-mut of DVL3, and subcellular localization of DVL3 and endogenous YAP was determined by confocal microscopy. Inset, DAPI; Scale bar, 5 μm.

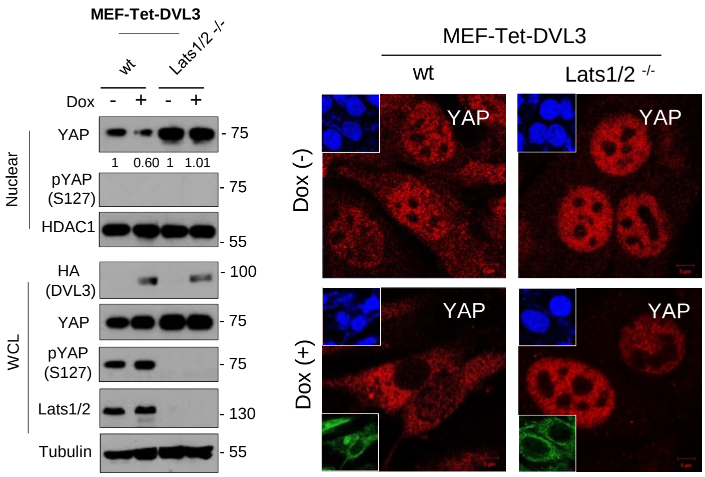

Supplementary Figure 6. Lats1/2 kinases-dependent YAP nuclear export by DVL. The wt and Lats1/2 double-knockout (Lats1/2$^{-/-}$) mouse embryonic fibroblast (MEF) cells were stably transfected with inducible DVL3, and nuclear YAP level was determined by immunoblotting (left panels) and confocal microscopy (right panels). Upper inset, DAPI; lower inset, HA (DVL3), Scale bar, 5 μm.

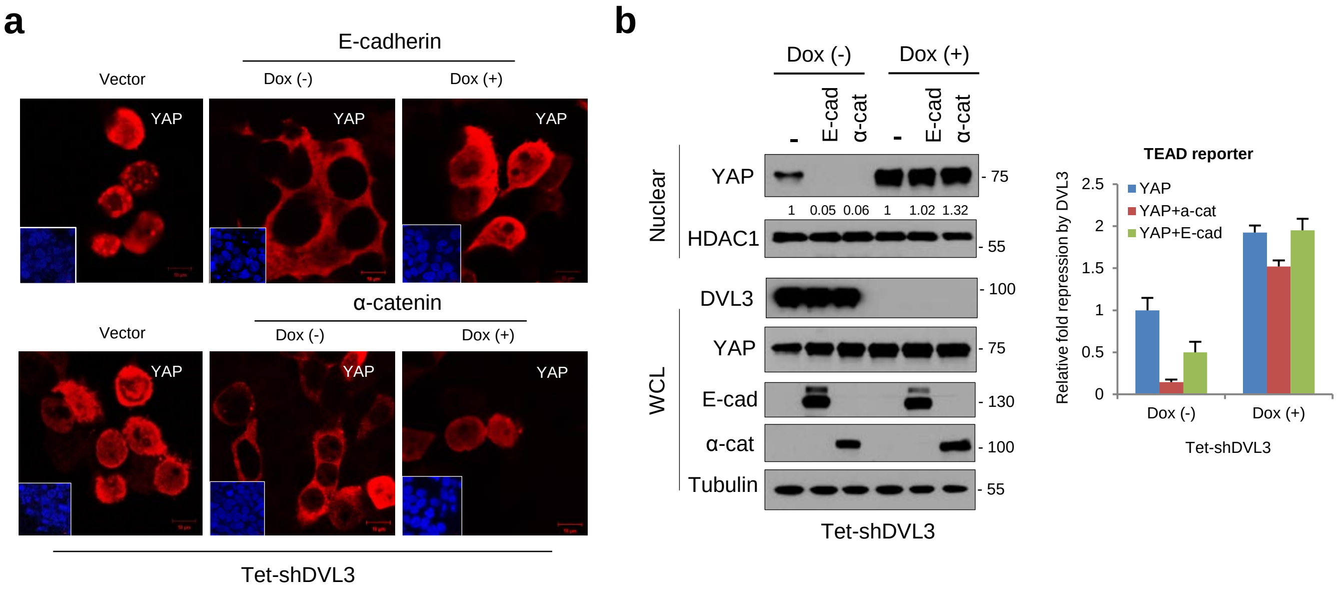

Supplementary Figure 7. Knockdown of DVL3 ablates YAP nuclear export. (**a**) The 293 cells expressing inducible shRNA for DVL3 were transfected with flag-tagged YAP (50 ng) in combination with vector control (1 μg) or E-cadherin (1 μg) or α-catenin (1 μg), and YAP localization was determined without (-) or with (+) doxycycline by confocal microscopy. Inset, DAPI nuclear stain; Scale bar, 10 μm. (**b**) The 293 cells were transfected with vector control (-) or E-cadherin (E-cad) or α-catenin (α-cat), and endogenous YAP abundance in nuclear fraction and whole cell lysates (WCL) was determined by immunoblot analysis (left panels). Relative nuclear YAP abundance compared to control was measured by ImageJ. The TEAD reporter was co-transfected with empty vector (-) or α-catenin (α-cat) or E-cadherin (E-cad) in control (Dox -) or inducible shRNA (Dox +) for DVL3 in 293 cells, and the reporter activity was measured (right panel). Data presented as mean ± SD, n = 3.

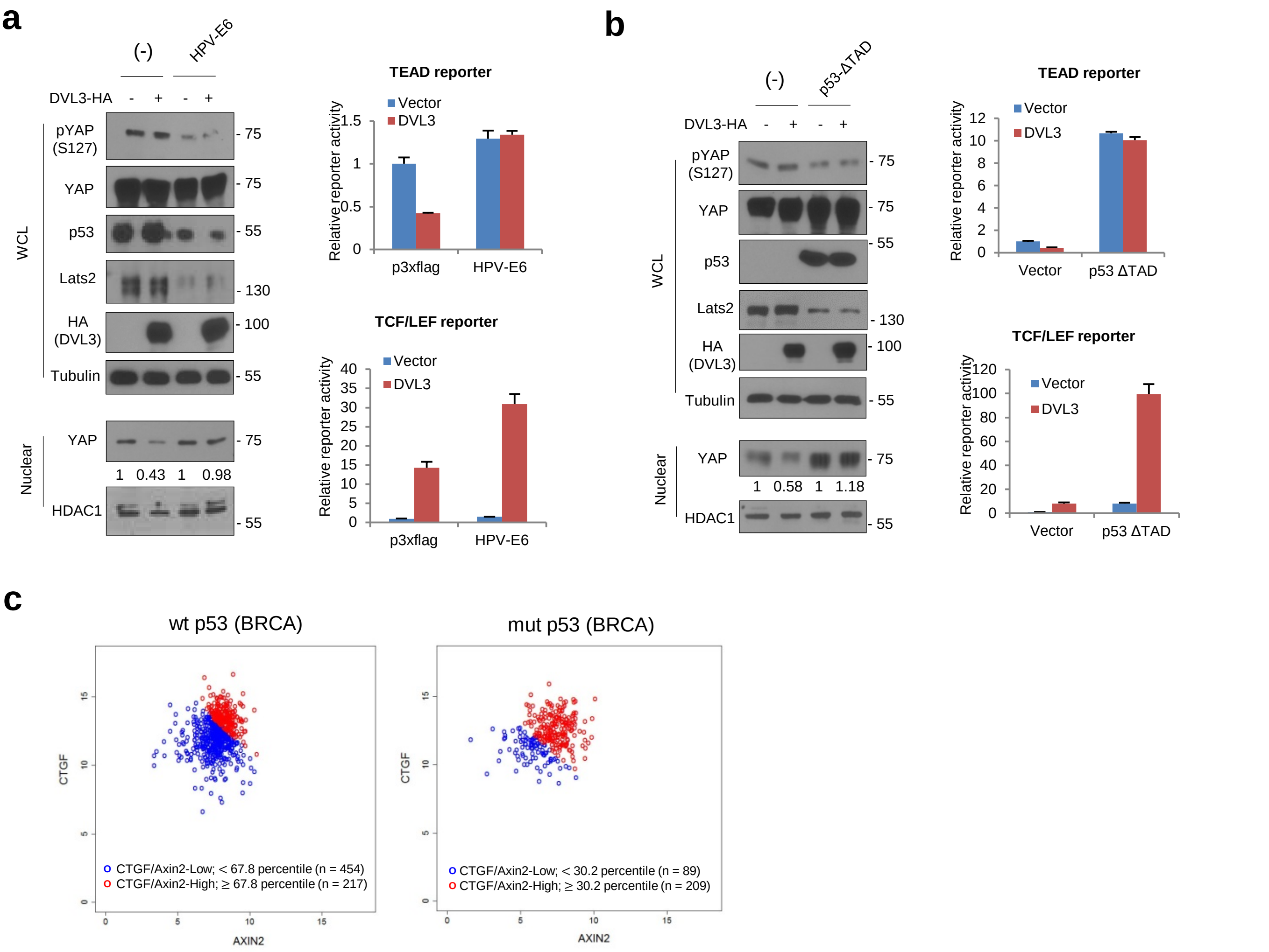

Supplementary Figure 8. Loss of p53 allows co-activation of nuclear YAP and canonical Wnt activities by DVL. (**a**) The 293 cells were transfected with control empty vector (-) or HPV-E6 expression vector in combination with HA-tagged DVL3. Whole cell lysates (WCL) were immunoblotted with anti-pYAP (Ser 127), anti-YAP, anti-Lats2, anti-p53, anti-HA (DVL3), and nuclear fractions were used for nuclear YAP abundance (left panels). TEAD (upper right) and TCF/LEF (lower right) reporter constructs were co-transfected with DVL3 in control transfected cell (-) or HPV-E6 transfected cells, and the relative reporter activity was measured from triplicate experiments. Data presented as mean ± SD. (**b**) The 293 cells were transfected with control empty vector (-) or p53 deleting transactivating domain (p53-ΔTAD) in combination with HA-tagged DVL3. Whole cell lysates (WCL) were immunoblotted with anti-pYAP (Ser 127), anti-YAP, anti-Lats2, anti-p53, and anti-HA (DVL3), and nuclear fractions were used for nuclear YAP abundance (left panels). TEAD (upper right) and TCF/LEF (lower right) reporter construct was co-transfected with DVL3 in control transfected cell (-) or p53-ΔTAD transfected cells, and the relative reporter activity was measured from triplicate experiments. Data presented as mean ± SD. (**c**) Scatter plots of CTGF and Axin2 transcripts in TCGA breast cancer samples (BRCA) according to p53 status. The high (CTGF-high/Axin2-high, red) and low (CTGF-low/Axin2-low, blue) subsets were determined based on the median transcript abundance of CTGF and Axin2 as described in Methods.

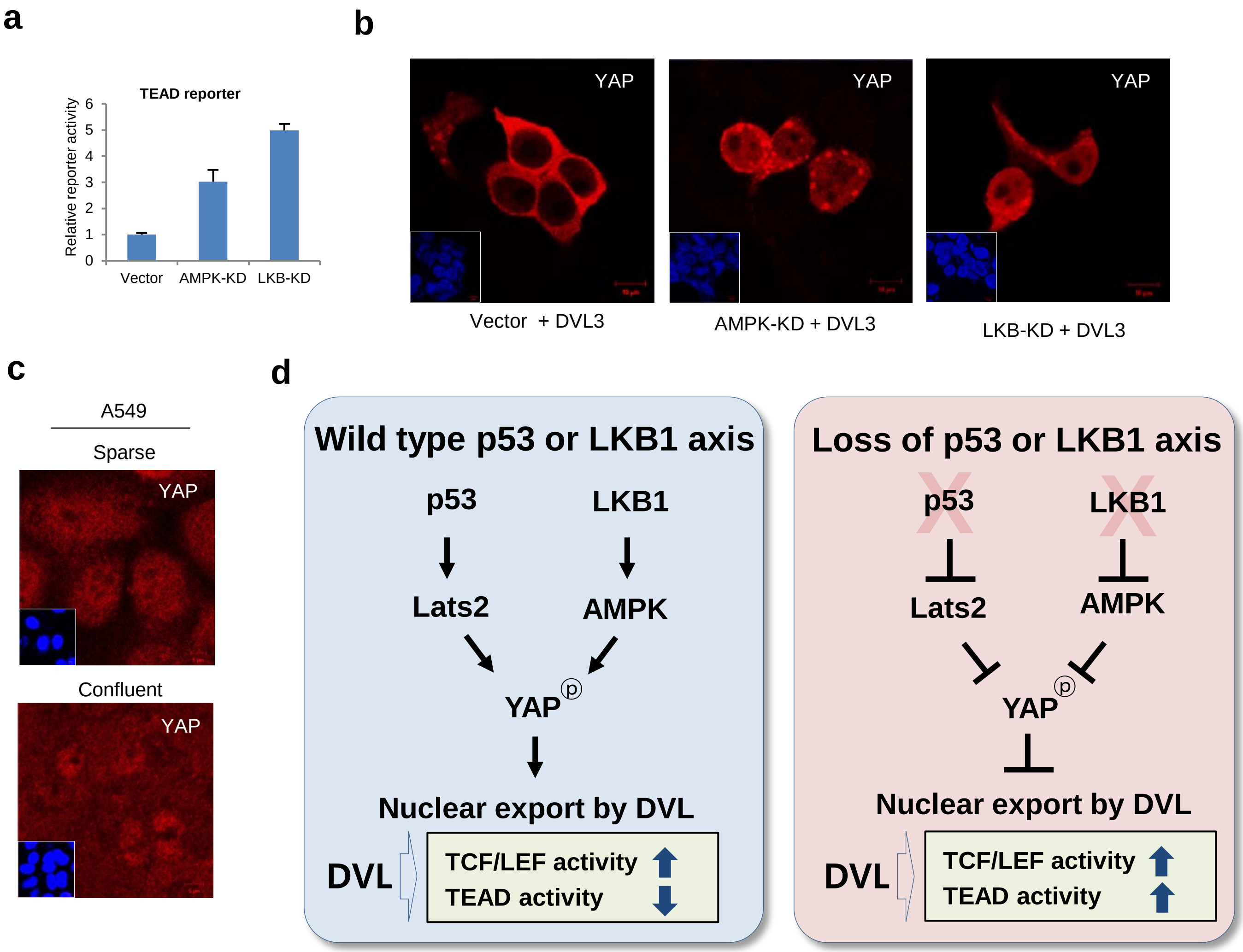

Supplementary Figure 9. Loss of LKB1/AMPK axis abolishes nuclear YAP export by DVL3. (**a**) Kinase-dead dominant negative AMPK (AMPK-KD) or LKB1 (LKB1-KD) increases TEAD reporter activity. The 293A cells were co-transfected with reporter construct having 8x TEAD binding sites in combination with vector control or AMPK-KD or LKB1-KD expression vector, and then relative reporter activity normalized by *Renilla* activity was measured by dual luciferase assay system. (**b**) The AMPK-KD or LKB1-KD was co-transfected with DVL3 and YAP localization was determined by confocal microscopy. Nuclear staining with DAPI is shown below each panel. Scale bar, 10 μm. (**c**) Dispersed intracellular localization of YAP in LKB1-deficient A549 cells. The A549 cells were cultured under sparse or confluent culture condition, and YAP localization was determined by confocal microscopy. The cells were serum starved for 16 hr before cell fixation procedure. Inset, DAPI; Scale bar, 5 μm. (d) A model for regulation of Hippo and canonical Wnt pathways by DVL. When tumour suppressor p53/Lats2 or LKB1/AMPK is functional, Wnt activator DVL suppresses nuclear YAP activity. Loss of p53 or LKB1 tumour suppressor axis disturbs YAP nuclear export by DVL allowing co-activation of canonical Wnt and nuclear YAP/TEAD activities.

Supplementary Figure 10 Uncropped images of all the western blot data

Fig. 1b

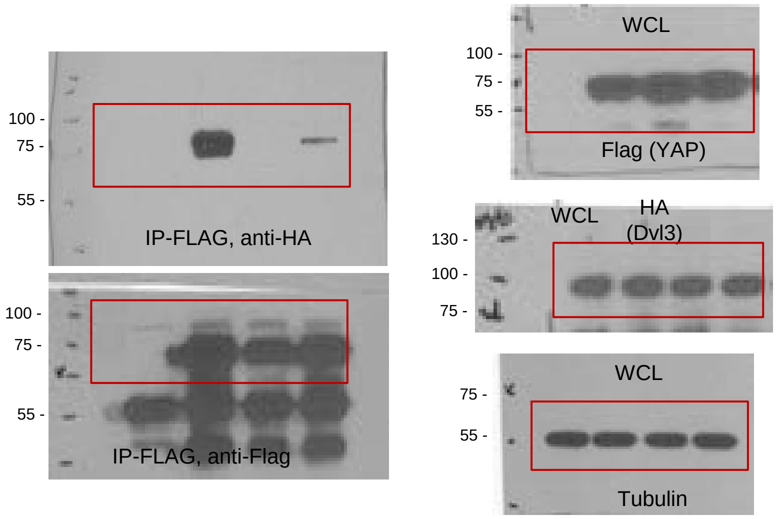

Fig. 1c

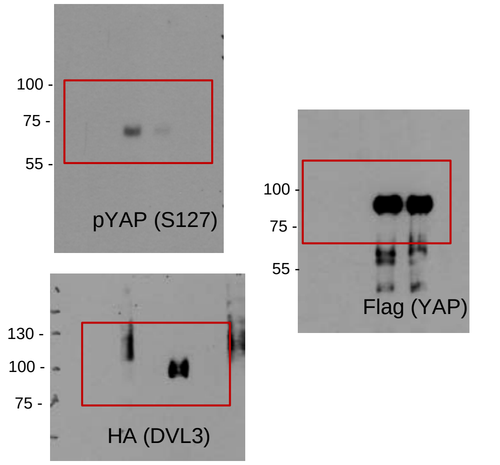

Fig. 1d

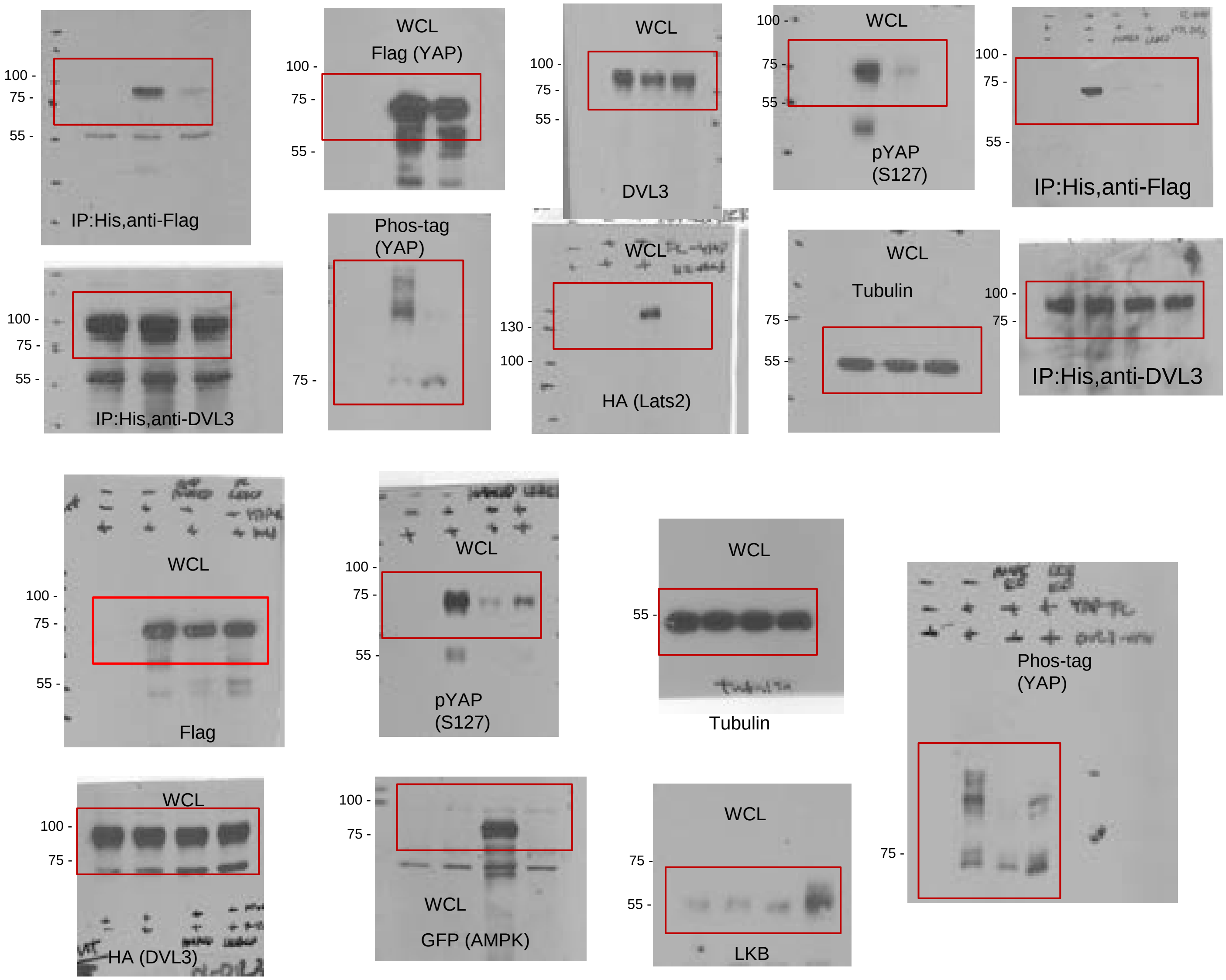

Fig. 1e

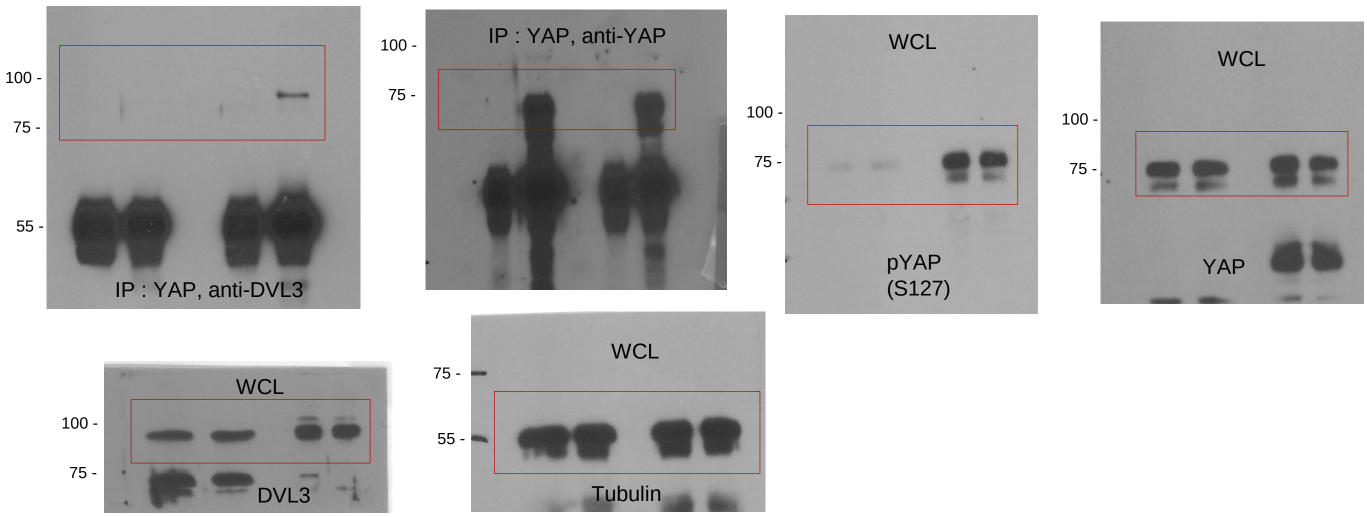

Fig. 2a

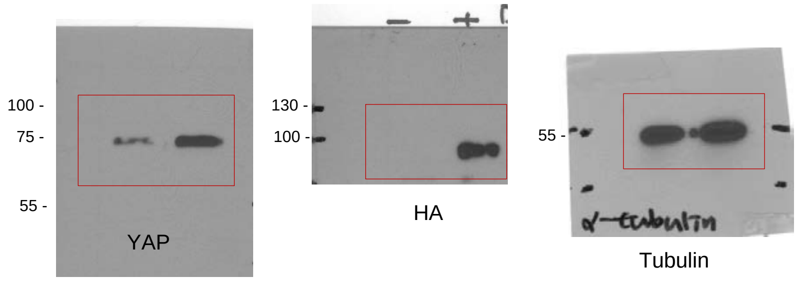

Fig. 2b

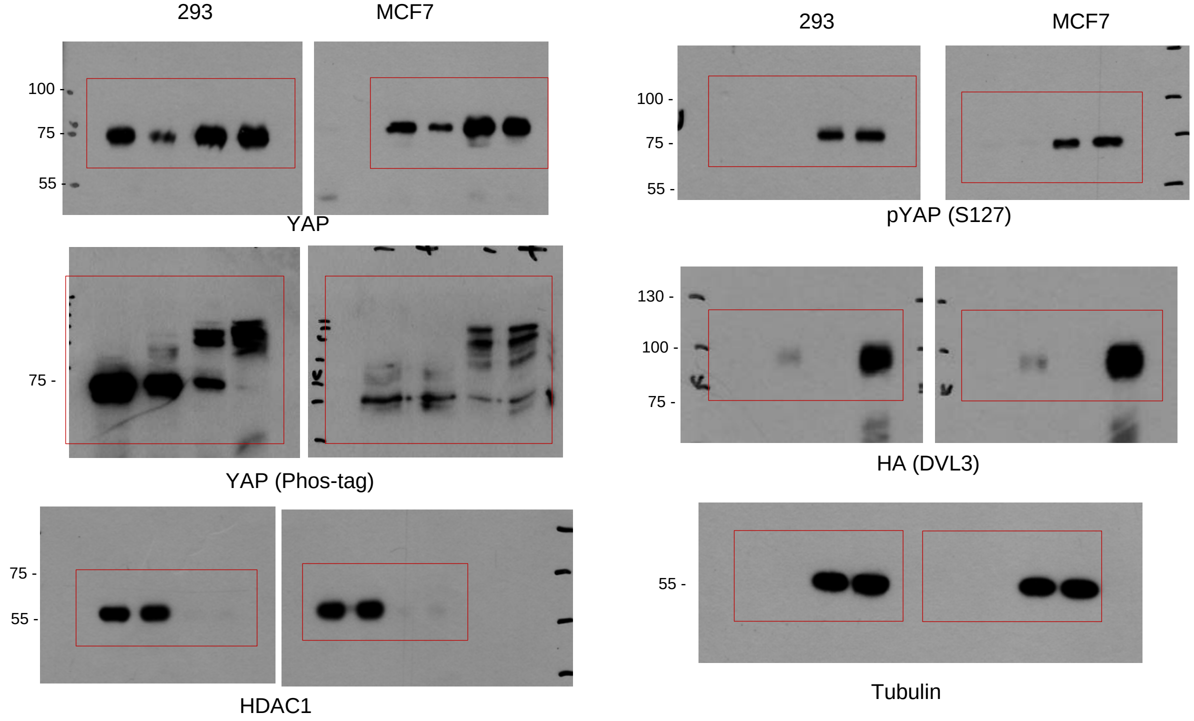

Fig. 2d

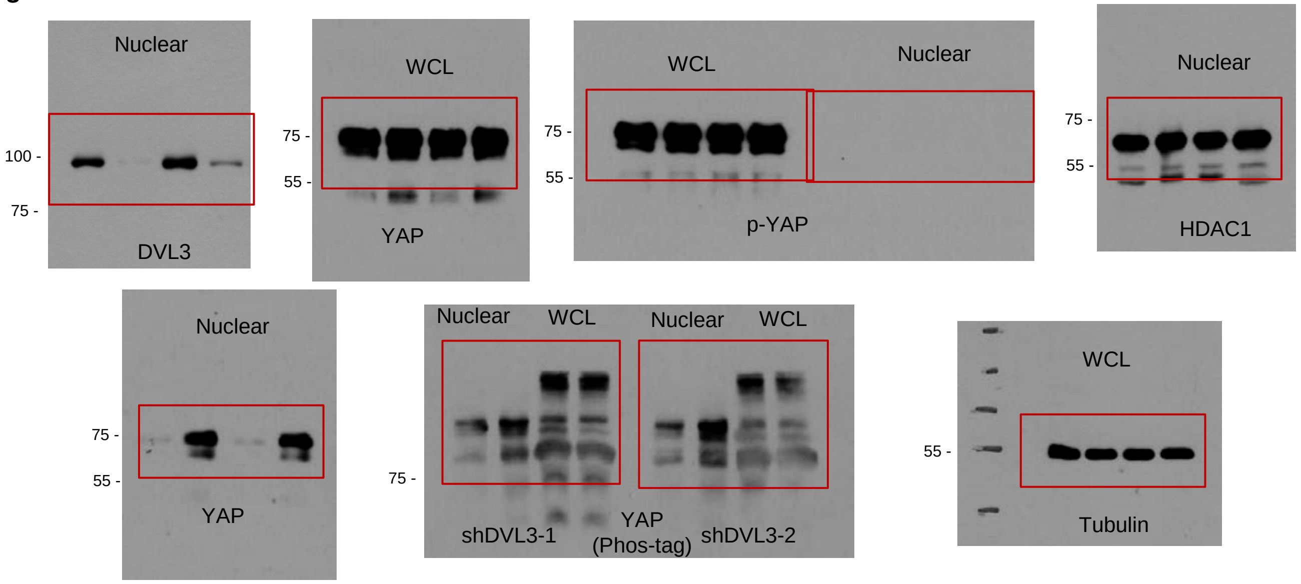

Fig. 2f

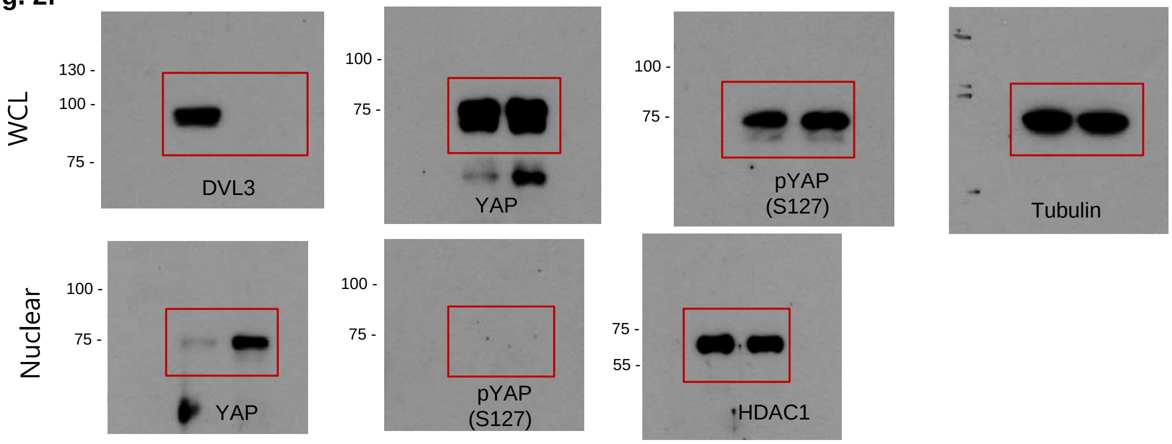

Fig. 3a

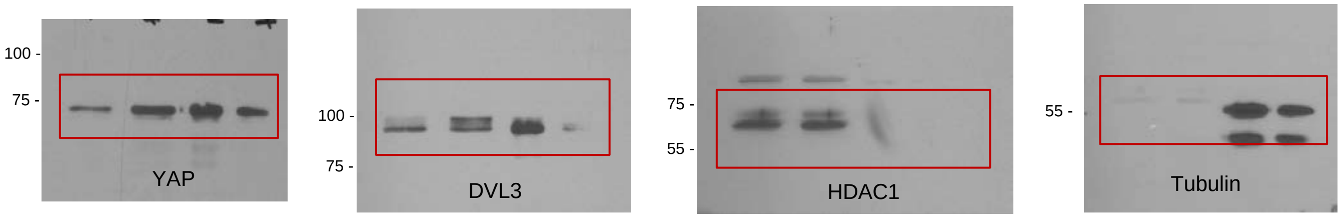

Fig. 3d

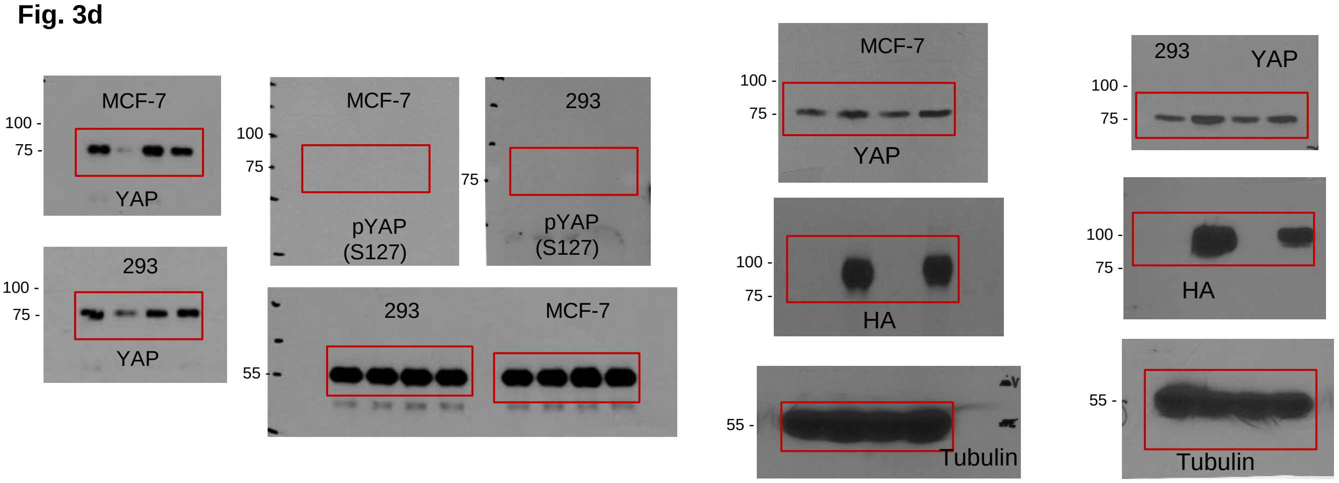

Fig. 3d (continued)

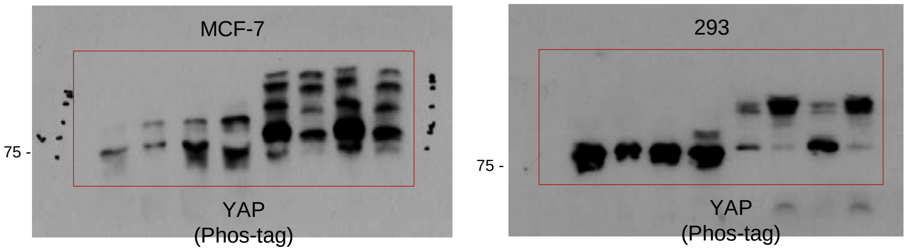

Fig. 3e

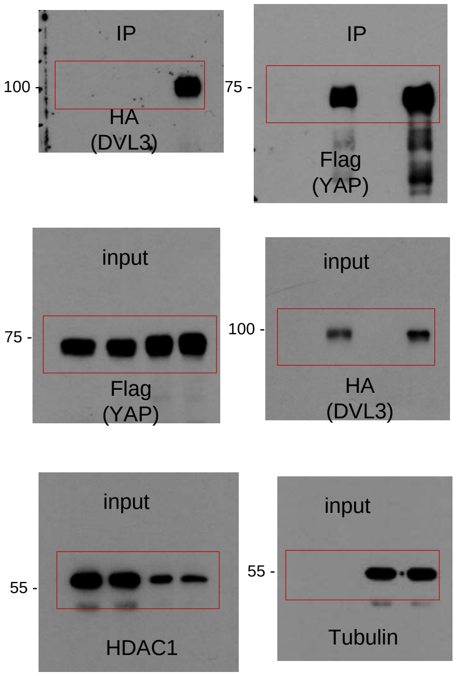

Fig. 4a

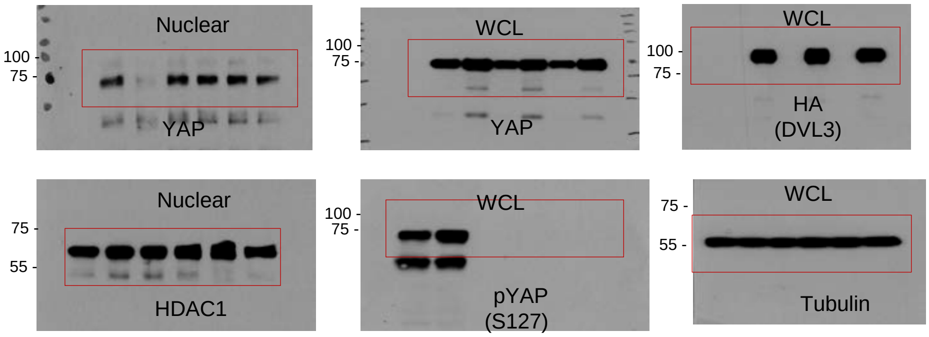

Fig. 4d

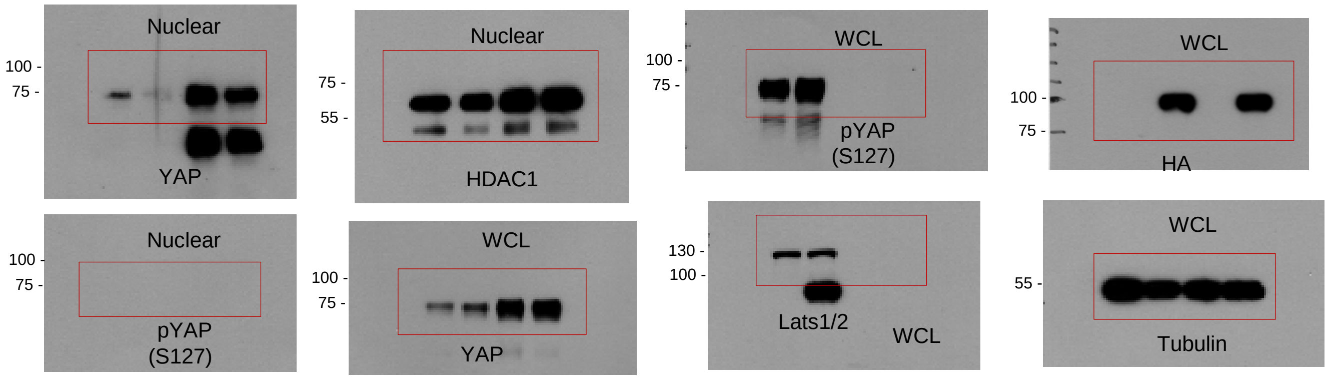

Fig. 4f

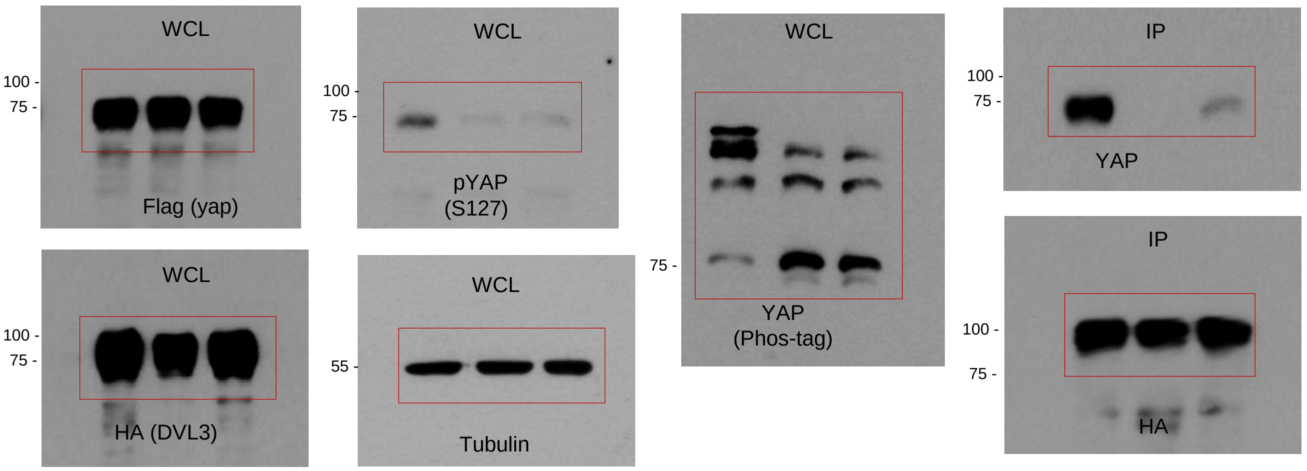

Fig. 5b

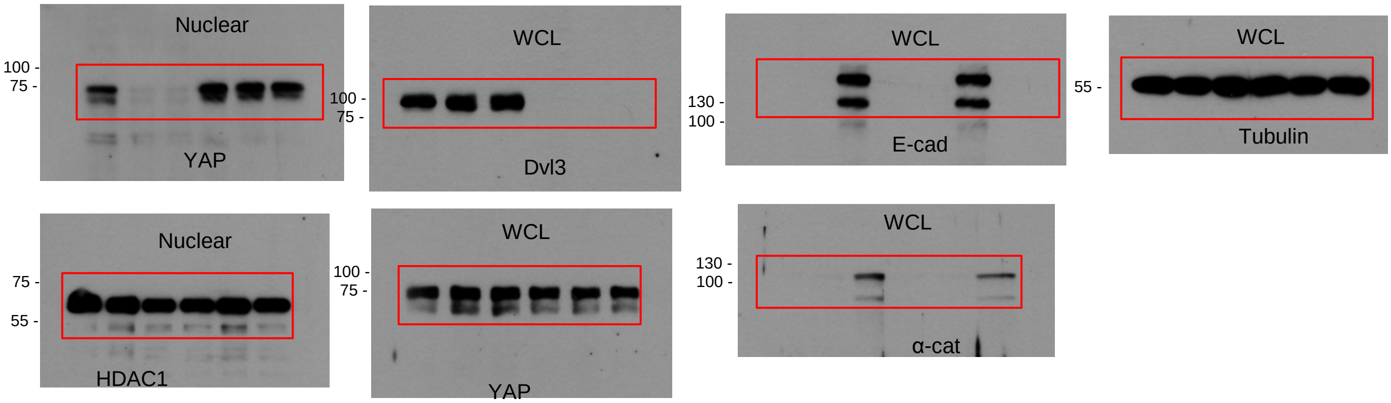

Fig. 6a

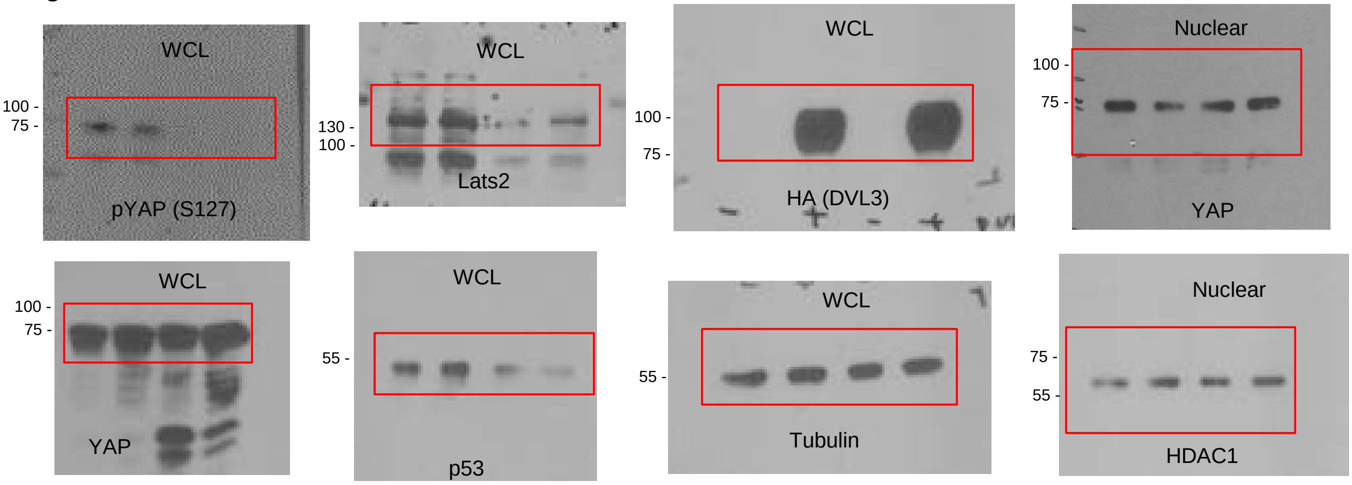

Fig. 6d

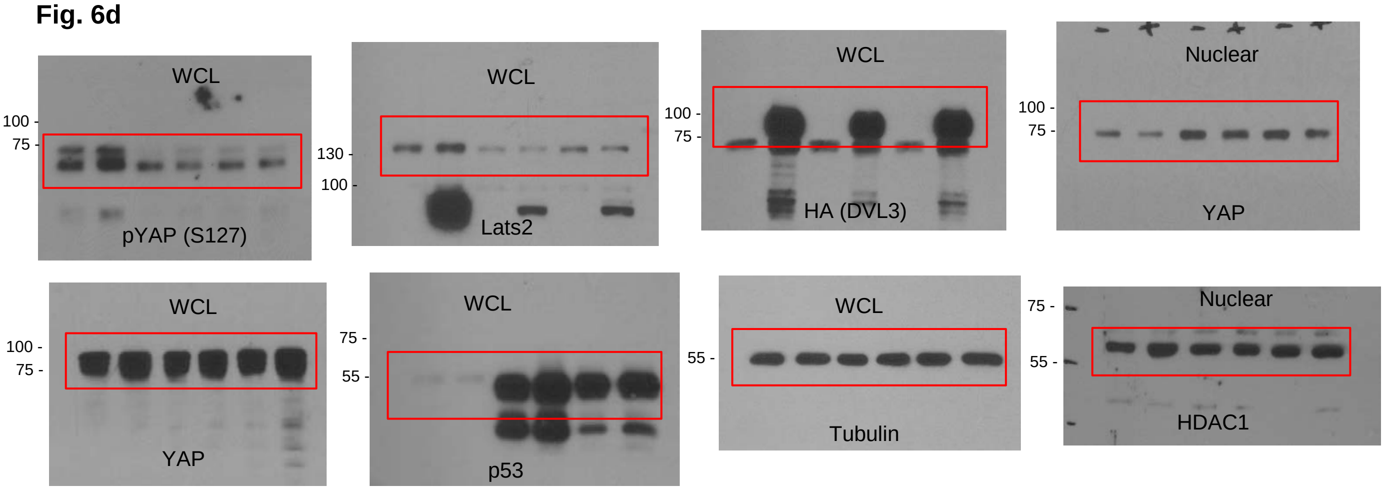

Fig. 7b

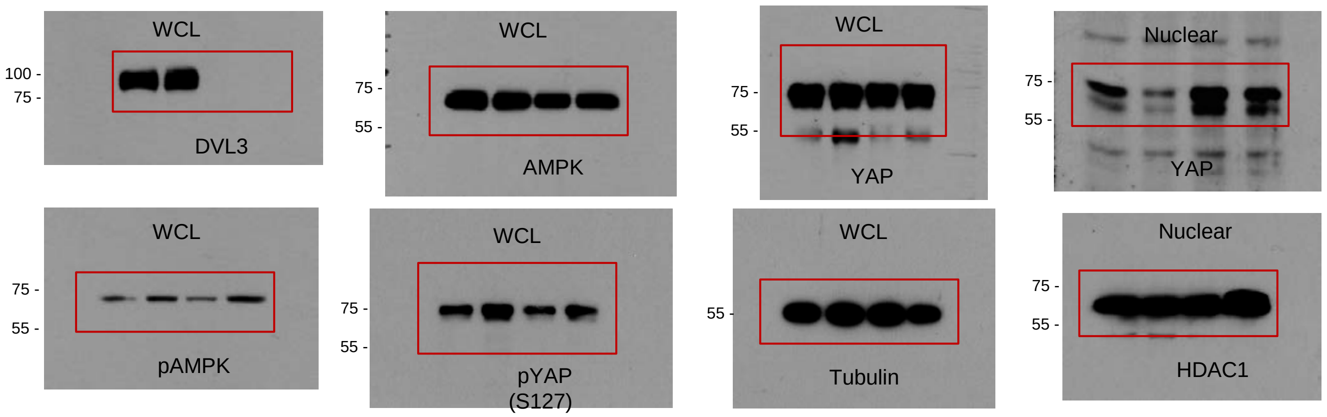

Fig. 7c

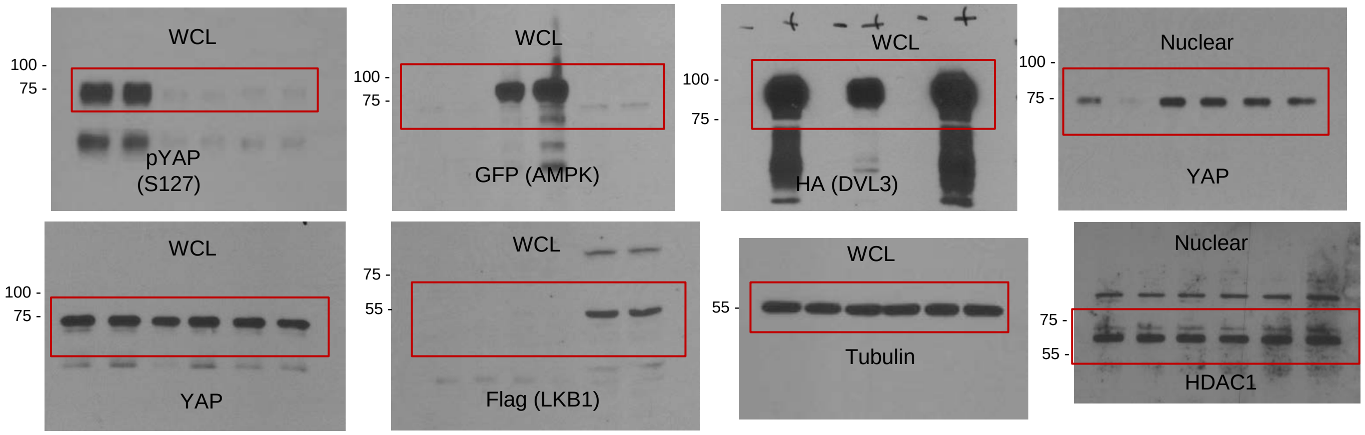

Fig. 7d

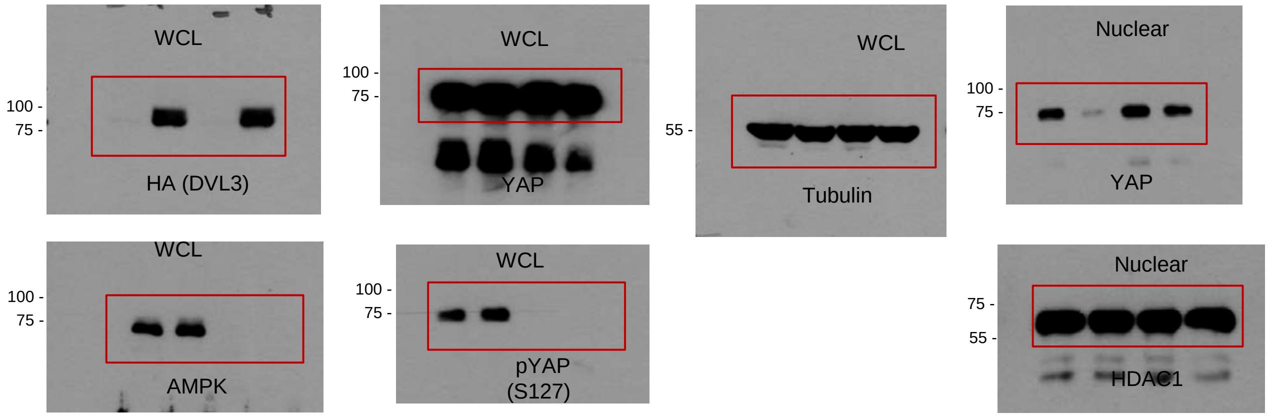

Fig. 7e

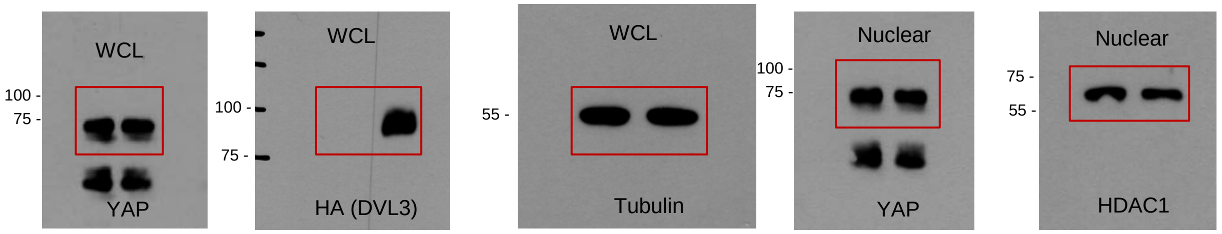

Supplementary Fig. 1b

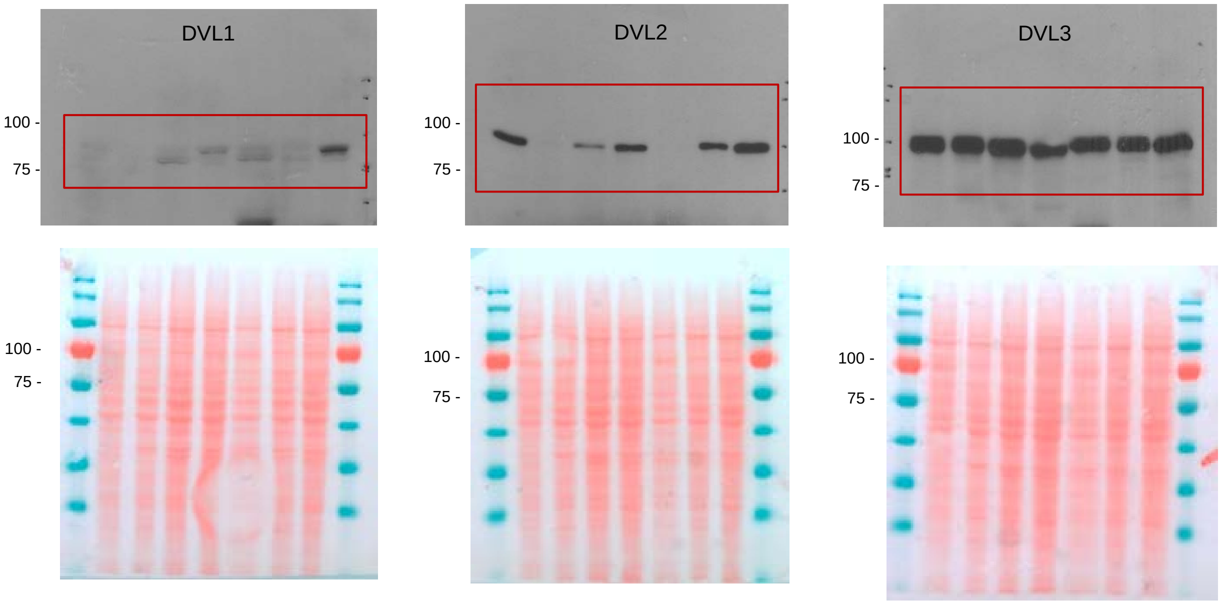

Supplementary Fig. 2a

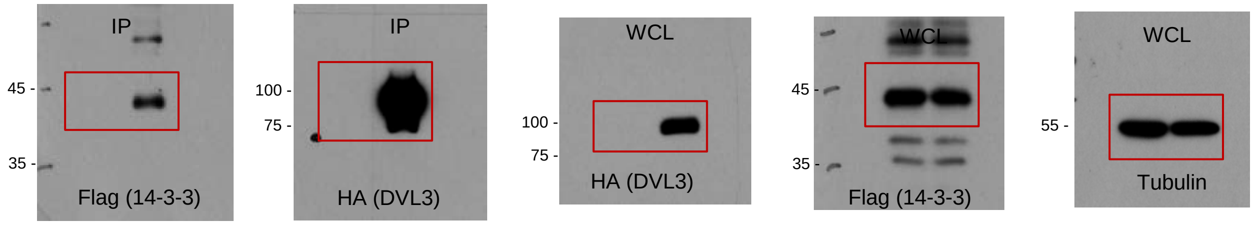

Supplementary Fig. 2b

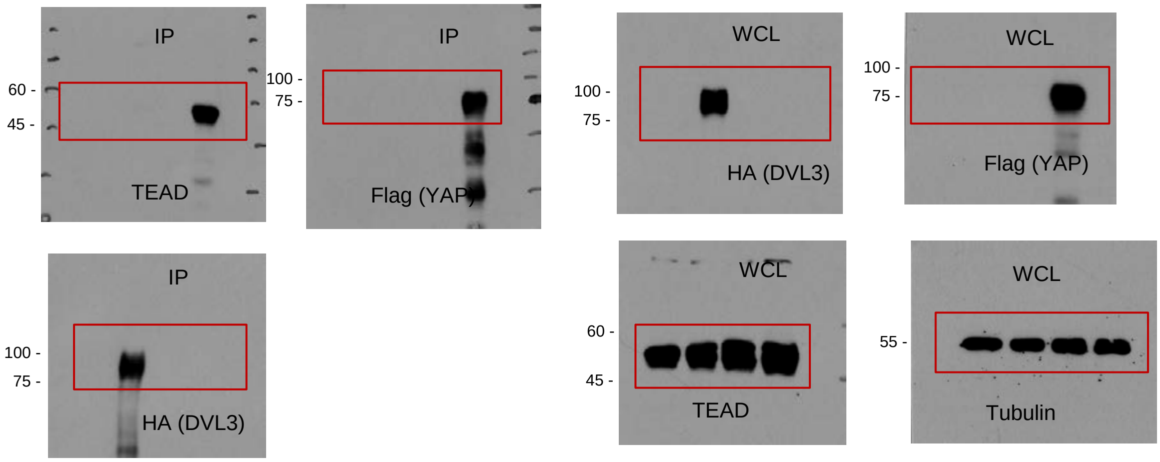

Supplementary Fig. 3a

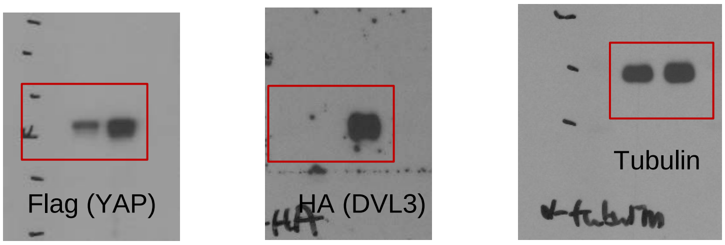

Supplementary Fig. 3c

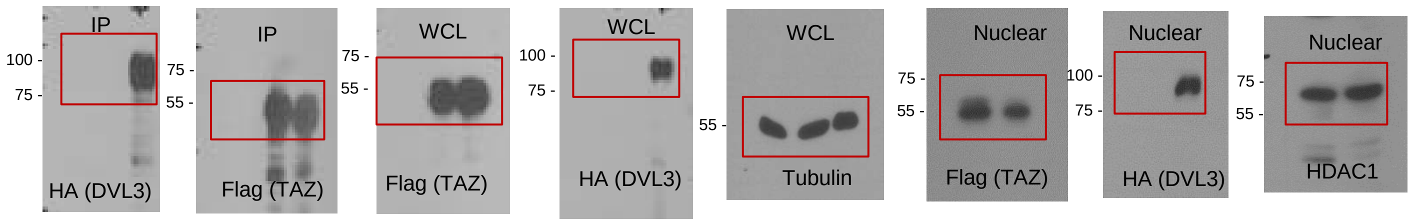

Supplementary Fig. 4a

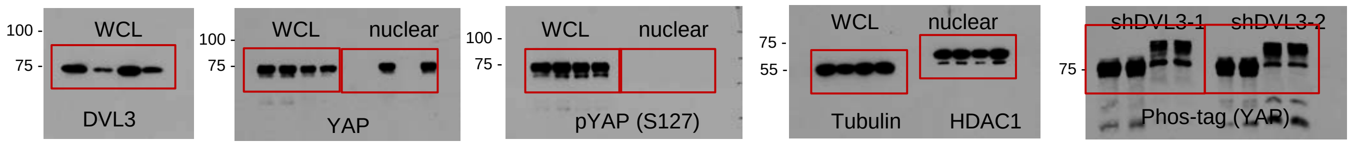

Supplementary Fig. 4b

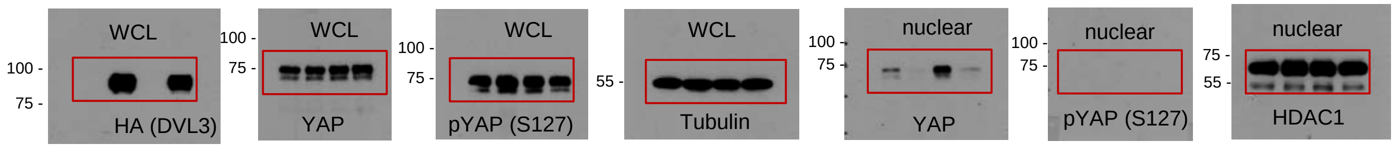

Supplementary Fig. 5a

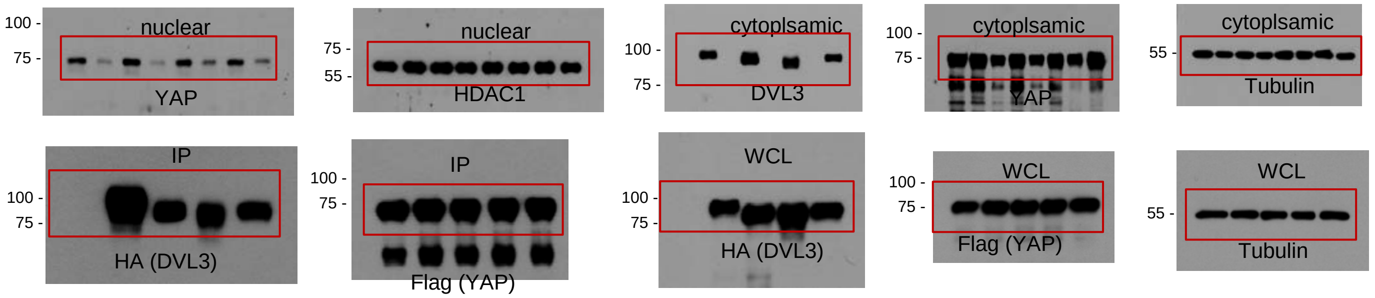

Supplementary Fig. 5b

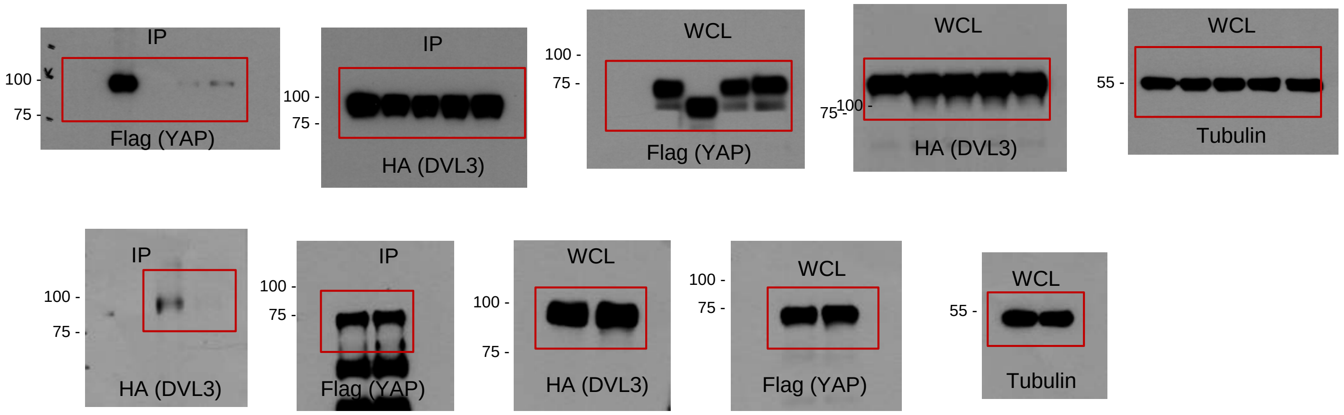

Supplementary Fig. 6

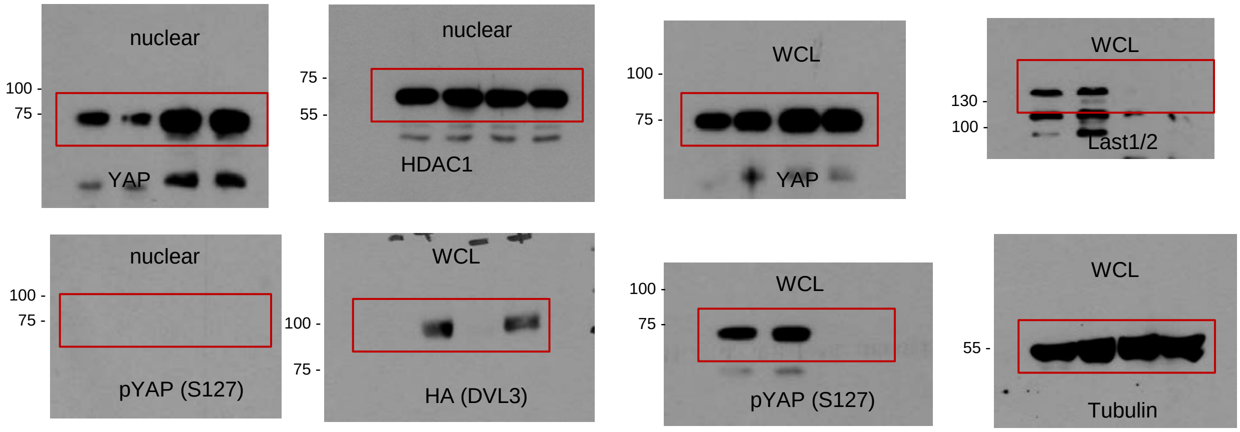

Supplementary Fig. 7b

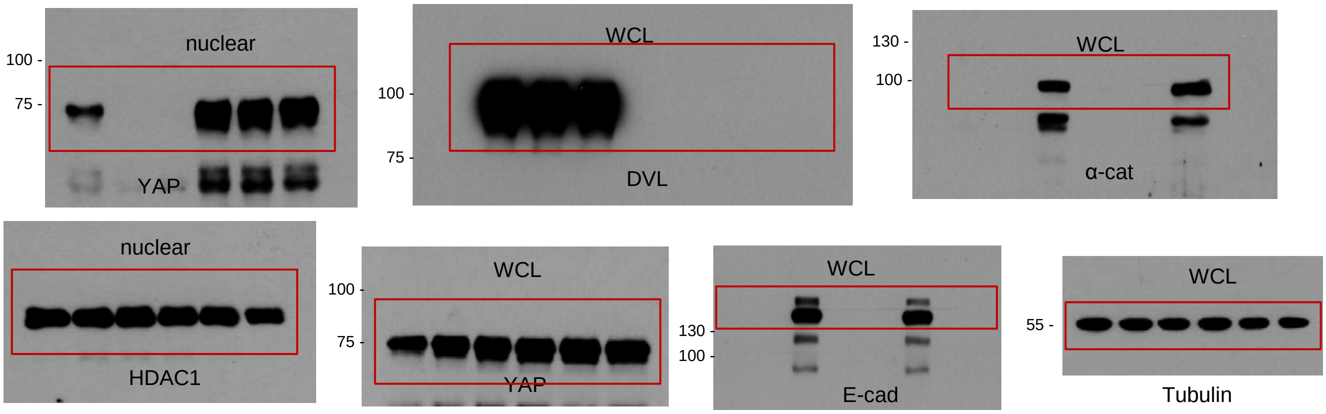

Supplementary Fig. 8a

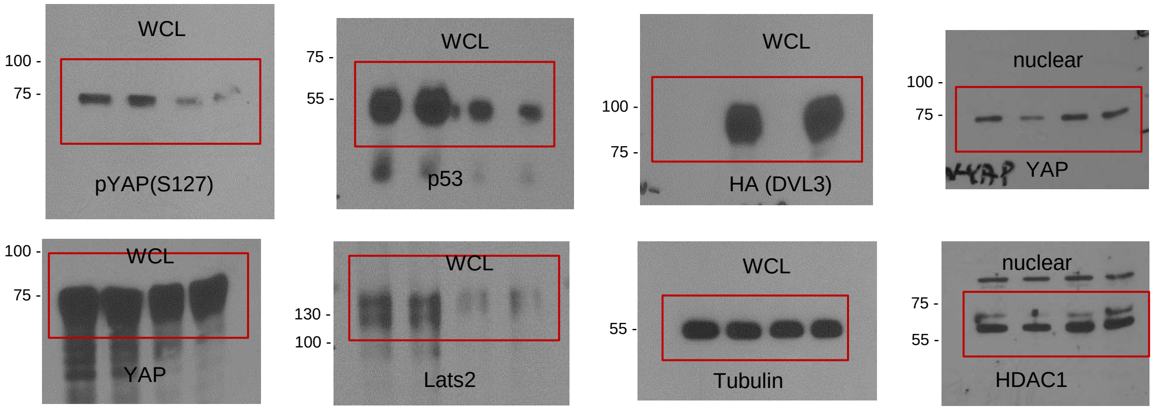

Supplementary Fig. 8b

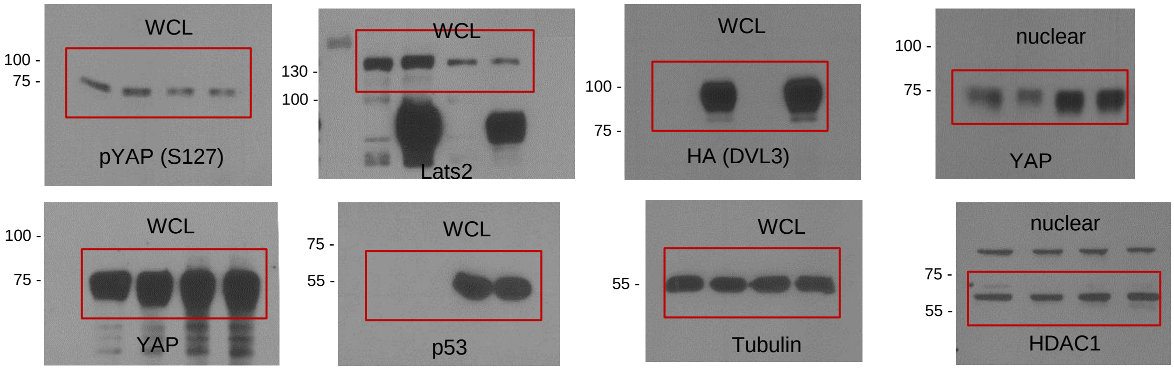

Supplement: Supplementary file 1 — Supplementary Information [file 41467_2018_4757_MOESM1_ESM.pdf]
